# [18F]fluorodeprenyl-D2 PET can detect and monitor astrogliosis in anti-LGI1-IgG autoimmune encephalitis

**DOI:** 10.1007/s00259-025-07531-5

**Published:** 2025-09-26

**Authors:** J. A. Gernert, L. Sanzo, H. Zimmermann, F. S. Thaler, L. Vogler, J. S. Gnörich, L. Tagnin, S. Lindner, B. Kugelmann, R. Schaefer, G. N. Bischof, S. Katzdobler, R. A. Werner, G. U. Höglinger, J. Levin, N. Franzmeier, B. Rauchmann, R. Perneczky, M. Kerschensteiner, T. Kümpfel, M. Brendel

**Affiliations:** 1 Institute of Clinical Neuroimmunology, LMU University Hospital, LMU Munich, Munich, Germany; 2 Biomedical Center, Medical Faculty, LMU Munich, Munich, Germany; 3 Department of Nuclear Medicine, LMU University Hospital, LMU Munich, Munich, Germany; 4 Institute of Neuroradiology, LMU University Hospital, LMU Munich, Munich, Germany; 5 Life Molecular Imaging GmbH, Berlin, Germany; 6 Department of Neurology, LMU University Hospital, LMU Munich, Munich, Germany; 7 German Center for Neurodegenerative Diseases (DZNE), Munich, Germany; 8 Russell H. Morgan Department of Radiology and Radiological Sciences, Johns Hopkins School of Medicine, Baltimore, MD US; 9 Munich Cluster for Systems Neurology (SyNergy), Munich, Germany; 10 The Sahlgrenska Academy, Institute of Neuroscience and Physiology, Department of Psychiatry and Neurochemistry, University of Gothenburg, Mölndal and Gothenburg, Gothenburg, Sweden; 11 Institute for Stroke and Dementia Research, LMU University Hospital, LMU Munich, Munich, Germany; 12 Department of Psychiatry and Psychotherapy, LMU University Hospital, LMU Munich, Munich, Germany; 13 Sheffield Institute for Translational Neuroscience (SITraN), University of Sheffield, Sheffield, UK; 14 Ageing Epidemiology Research Unit (AGE), School of Public Health, Imperial College London, London, UK

**Keywords:** Anti-LGI1-IgG autoimmune encephalitis, [^18^F]fluorodeprenyl-D2 PET-CT, Neuroinflammation, Astrocytosis

## Abstract

**Purpose:**

To explore whether detecting local astrogliosis using [^18^F]fluorodeprenyl-D2 ([^18^F]F-DED) positron-emission-tomography (PET) uncovers and monitors inflammatory lesions in patients with anti-leucine-rich glioma-inactivated 1 antibody (LGI1-ab) associated autoimmune encephalitis (AE).

**Methods:**

Dynamic [^18^F]F-DED PET scans (0–60 min post-injection) were obtained from a cohort of 15 LGI1-AE patients, 9 of whom were re-examined during the disease course, and from 15 controls. PET quantification was performed by kinetic modelling with an image derived input function and calculation of simplified standardized uptake values (SUV). [^18^F]F-DED SUVr (referenced to the straight gyrus) were analyzed for LGl1-AE target regions, compared between baseline and follow-up PET scans, used to investigate asymmetry of the mesial temporal lobe (MTL), and correlated to routine clinical data.

**Results:**

Simplified [^18^F]F-DED PET quantification of the 30–60 min time-frame showed excellent agreement with kinetic modelling of the full dynamic imaging protocol. In LGI1-AE patients, [^18^F]F-DED SUVr values were significantly increased by 16% in the MTL (*p* = .002) and by 12% in the cerebellum (*p* = .014). [^18^F]F-DED signals in the MTL significantly declined during the course of the disease (mean follow-up time: 15 months, *p* = .006). MTL asymmetry (> 5%) of [^18^F]F-DED SUVr was present in 8/15 of LGI1-AE patients compared to 1/15 controls (*p* < .001) and showed marked decline between baseline and follow-up in LGI1-AE patients.

**Conclusion:**

This pilot study shows that [^18^F]F-DED PET is a promising tool to monitor regional astrogliosis in LGI1-AE patients and may provide a direct read-out of this important aspect of inflammatory disease activity.

**Supplementary Information:**

The online version contains supplementary material available at 10.1007/s00259-025-07531-5.

## Introduction

Anti-leucine-rich glioma-inactivated 1 (LGI1) antibody-associated autoimmune-encephalitis (AE) is the second most common form of autoimmune encephalitis that primarily affects elderly men and mainly presents with limbic encephalitis often preceded by faciobrachial dystonic seizures (FBDS) [1]. About 25% of patients experience relapses during the course of disease which are typically milder than the initial manifestation but still cause additional disability [2]. Therefore, monitoring disease activity in subjects with LGI1-AE is crucial. Beyond clinical examinations and re-testing of antibody (ab) titers, neuroimaging using magnetic resonance imaging (MRI) and positron-emission-tomography (PET) has been evaluated for disease surveillance. LGI1-AE related pathologies on MRI are stage-depended: Mesiotemporal hyperintensities on T2-weighted (T2w) and Fluid-Attenuated Inversion Recovery (FLAIR) -MRI are a typical feature (60–70%) in the acute stage [3]. However, MRI presentation is unsuspicious at this stage, especially in persons with FBDS manifestation [3–5]. Follow-up MRI frequently reveals hippocampal atrophy [3, 4]. In the last decade different PET-radiotracers have been evaluated to assess distinct pathological aspects in LGI1-AE cohorts. 2-Deoxy-2-[^18^F]fluoro-D-glucose (FDG-PET) neuroimaging repeatedly demonstrated hypermetabolism in the hippocampus and basal ganglia [6]. Further, glucose metabolism, assessed by FDG-PET, indicated disease activity more sensitively than MRI especially in patients with FBDS and might therefore be a complementary examination to support treatment decisions [7–9]. As a more direct read-out of cerebral inflammation, the 18 kDa translocator protein (TSPO) ligand [^18^F]DPA-714 was used as a proxy of microglial activation and PET imaging of LGI1-AE patients detected altered signal patterns associated with distinct clinical symptoms [10]. Finally, [^18^F]florbetapir- and [^18^F]flortaucipir PET imaging for assessment of β-amyloid and tau aggregation showed [^18^F]flortaucipir PET retention in individual patients [11, 12].

Despite these approaches detecting and monitoring lesion pathology and disease activity in LGl1-AE remains challenging not the least because the abovementioned changes to CNS metabolism or microglia activation states are often transient. Here the detection of lesion-associated astrogliosis offers an attractive opportunity as the reactive changes to astrocytes are induced during initial autoimmune lesion formation and then persist long-term as astroglial scars, which can presumably be prevented by immunotherapy [13]. Thus, imaging approaches that monitor astrogliosis are expected to not only detect lesions during their acute inflammatory stage but also the remaining disease activity in scars of the lesions that have formed previously. Indeed, persistent astrogliosis has been described in LGI1-AE cases with histopathological examination [14–16]. In this regard, PET imaging using the radiotracer [^18^F]fluorodeprenyl-D2 ([^18^F]F-DED) has emerged as a novel method to evaluate regional astrogliosis in vivo based on the expression of the monoamine oxidase B (MAO-B) [17]. MAO-B is primarily expressed on mitochondrial membranes in glial cells and its expression is increased during reactive astrogliosis [17].

Thus, we analyzed [^18^F]F-DED PET scans in patients with LGI1-AE to cross-sectionally compare [^18^F]F-DED binding with a control cohort and to longitudinally assess tracer uptake in the disease course of patients with LGI1-AE. We aimed to investigate if [^18^F]F-DED PET (i) can detect astrogliosis in patients with LGI1-AE, (ii) allows monitoring of changes in MAO-B expression as a proxy of astroglial disease activity, (iii) can provide complementary information compared to MRI and clinical assessment.

## Materials and methods

### Study design and cohorts

We included subjects with LGI1-AE diagnosed according to current criteria [18] that received [^18^F]F-DED PET for diagnostic purpose at LMU Hospital Munich between 10/2021 and 07/2024. LGI1–ab testing in serum and cerebrospinal fluid (CSF) was performed by cell-based assay used in clinical practice (EUROIMMUN Medizinische Labordiagnostika AG, Lübeck, Germany). Patients were consecutively seen and followed-up by neurologists specialized in the treatment of AE from the Institute of Clinical Neuroimmunology, LMU Hospital. Clinical data, including Montreal Cognitive Assessment (MoCA) and the modified Rankin score (mRS), as well as laboratory results including ab titers in serum and CSF were collected in a standardized manner. The in-house control cohort (*n* = 15) included thirteen individuals without objective memory deficits (SCD; all with negative β-amyloid status), one patient with a small PET-negative oligodendroglioma in the frontal subcortical white matter and one younger individual with no objectified diagnosis (for detailed description please refer to Table 1). The study was conducted in accordance with the principles of the Declaration of Helsinki. All subjects included gave written informed consent. The local ethical committee approved this study (IRB numbers: 21–0721, 22–0997). Imaging of controls was waived by the German radiation protection authorities (BfS: ZD 3-22464/2023-042-G).

**Table 1:** Overview of LGI1-AE cohort at initial disease manifestation

	P1	P2	P3	P4	P5	P6	P7	P8	P9	P10	P11	P12	P13	P14	P15
**Clinical data**															
Gender	female	male	male	male	male	male	female	male	male	male	female	male	male	female	male
Age at IM, years	47	68	73	70	56	57	64	67	53	58	76	62	74	64	55
Presenting features	Epileptic seizure, cognitive impairment, insomnia	Epileptic seizures, FBDS, cognitive impairment, hyponatremia	Epileptic seizure, FBDS, cognitive impairment, psychiatric symptoms	Epileptic seizure, cognitive impairment	Epileptic seizure, cognitive impairment	Cognitive impairment, psychiatric symptoms	FBDS, neuro-psychological abnormalities	Cognitive impairment, insomnia, ataxia	Epileptic seizure, cognitive impairment, psychiatric symptoms	Epileptic seizure, cognitive impairment, psychiatric symptoms, hyponatremia	Cognitive impairment, psychiatric symptoms	Epileptic seizure, cognitive impairment, hyponatremia	FBDS, cognitive impairment, insomnia	Epileptic seizure, cognitive impairment, hyponatremia	Generalized myoclonus, cognitive impairment, psychiatric symptoms
MoCA score (maIM)	14/30 (0)	13/30 (0)	21/30 (3)	29/30 (0)	na	25/30 (0)	na	25/30 (3)	27/30 (23)	23/30 (5)	14/30 (1)	na	na	16/30 (19)	na
LGI1-Ab titer (maIM)															
Serum (maIM)	1:200 (0)	1:1600 (0)	1:400 (3)	1:1600 (0)	1:20 (1)	1:800 (0)	1:100 (7)	1:400 (1)	1:200 (24)	1:400 (4)	1:800 (1)	detectable *	1:400 (6)	1:100 (20)	detectable *
CSF (maIM)	1:50 (0)	1:100 (0)	1:1 (3)	1:5 (0)	nd (1)	1:5 (0)	nd (7)	1:5 (1)	nd (24)	1:50 (4)	1:10 (1)	detectable *	1:10 (6)	1:3.2 (20)	detectable *
Other Ab titer detectable	Caspr2														NMDA
IM to Immunotherapy, months	0	0	2	0	0	0	9	1	23	5	1	0	7	17	6
Immunotherapy at initial manifestation	Corticosteroids PLEX IVIG Rituximab	Corticosteroids PLEX Rituximab	Corticosteroids PLEX Rituximab	Corticosteroids PLEX	Cortico-steroids	Corticosteroids PLEX Rituximab	Corticosteroids	Corticosteroids PLEX Rituximab	Corticosteroids MTX	Corticosteroids PLEX IVIG Rituximab	Corticosteroids PLEX IVIG MTX	Corticosteroids IVIG	Corticosteroids PLEX Rituximab	PLEX Cyclophosphamid	Corticosteroids Azathioprin

Ab: antibody; Caspr2: Contactin-associated protein-2; CSF: cerebrospinal fluid; FBDS: faciobrachial dystonic seizures; IM initial manifestation; IVIG: intravenouse immunglobuline; maIM: months after initial manifestation; MoCA: Montreal-Cognitive-Assessment; MTX: methotrexate; na: not available; nd: not detectable; NMDA: N-Methyl-D-Aspartat; PLEX: plasmapheresis

* External testing, no further information available

### MRI evaluation

In addition to the analysis of the PET images, the clinical routine MRI scans of the LGI1-AE cohort were evaluated. MRI scans were performed on different scanners but allowed standardized qualitative evaluation of (i) white matter hyperintensities, (ii) atrophy or swelling and (iii) contrast enhancement (available in 11/15). The mesial temporal lobe and the basal ganglia were evaluated as predilection sites of MRI signal alterations known from the literature using T2w/FLAIR and T1w sequences with and without contrast agent (Gd) administration with a maximum slice thickness of 3 mm [3–5]. A dichotomous visual read was performed by an expert in neuroradiology (H.Z.).

### [^18^F]F-DED-PET imaging

[^18^F]F-DED was synthesized on a Trasis AllinOne (Ans, Belgium) automated synthesis unit (ASU) consisting of 3 series-connected manifolds with a total of 18 valves as described previously [17]. In brief, the product was obtained in a RCY of 15 ± 3.0% n.d.c. (*n* = 16) and a RCP of 98 ± 1.2% (*n* = 16). Specific activity was 267 ± 120 GBq/µmol (*n* = 7). Dynamic [^18^F]F-DED PET scans (0–60 min post-injection, ~ 184 MBq) were performed between 10/2021 and 07/2024 at the Department of Nuclear Medicine, LMU Hospital, acquired and analyzed as described previously [17]. In brief, spatial normalization via structural MRI scans was achieved using PMOD (V4.3, PMOD Technologies GmbH, Faellanden, Switzerland). Volumes of distribution (VT) were determined using a one-tissue-compartment model (1TC2k) with a carotid image derived input function [17], as previously established for tau-PET imaging [19]. For simplified quantification, standardized uptake values (SUV) were extracted form a late time window (30–60 min post-injection) and referenced to the straight gyrus (SUVr), since previous autoradiography studies demonstrated high physiological MAO-B expression also in the frontal cortex [20]. Furthermore, the straight gyrus rectus was not identified as predilection site in LGI1-AE in an MRI meta-analysis and a comparative analysis [5, 21], likely due to its low LGI1 expression [5]. As a validation analysis, reference tissue modelling was performed using the simplified reference tissue model 2 (SRTM2) in PMOD. The straight gyrus served as the reference tissue to calculate distribution volume ratios (DVR), where the mesial temporal lobe (MTL) served as the target tissue. Predefined target regions were evaluated using the Hammers atlas in PMOD [22]: MTL, cerebellum, and basal ganglia as typical AE target regions as well as parietal and occipital lobe as target regions of global changes in astrogliosis. For follow-up analyses we additionally performed a manual delineation of the MTL and applied the equal region of interest to the follow-up scan. For this purpose, we used a clinical toolkit (Hermes Brass, Hermes Medical Solutions, Stockholm, Sweden). A non-blinded visual read that evaluated tracer uptake in the MTL (equal, mild/moderate/severe elevation compared to controls) was performed in clinical routine by one expert in molecular imaging (M.B.). To compare [^18^F]F-DED PET results with MRI findings in the MTL, we assessed [^18^F]F-DED uptake in the right and left MTL both with the asymmetry index (AI > 5%) and with a z-score > 2, categorizing patients as having either abnormal or normal uptake. Patients were classified as having an overall abnormal [^18^F]F-DED PET (DED overall) if either MTL showed abnormal uptake.

### Statistical analysis

For each target VOI, a Shapiro-Wilk test was executed to assess the normality of the data, followed by a Bartlett test to examine the homogeneity of variances. If both tests showed no significant results across all target regions, an independent two-sample t-test was performed. If normal distribution was not met, a Wilcoxon rank-sum test was calculated. In case of multiple testing, an FDR correction was applied. For baseline versus follow-up comparisons, we used two-sided paired t-tests, preceded by testing the differences for normality. The asymmetry index (AI) was calculated using the formula: AI = [200x(L-R)/(L + R)] %. To calculate z-scores for each patient, SUVr values from individual LGI1-AE and controls were standardized using the mean and standard deviation (SD) derived from the control group. Furthermore, correlations were investigated using a linear regression model and the Spearman’s rank correlation coefficient, as the assumption of normal distribution was not met. A p-value of *p* <.05 was regarded as significant. All descriptive and inference statistics were computed using R Core Team (2022).

## Results

### Study population

In total, 15 patients with LGI1-AE (4 female, 11 male) with a mean age of 67 ± 9 years at initial manifestation were included. Please refer to Table 1 for demographic and clinical data of the LGI1-AE cohort at their initial manifestation. Data for the control cohort is displayed in Supplement Table 1. Gender (*p* =.26) and age (*p* =.18) were not significantly different between both cohorts. Patients were investigated at different disease stages: Two patients were scanned within the first month of the initial manifestation (P1, P2), while P13 and P14 were examined within 6 months of a relapse with clinical deterioration and re-occurrence of LGI1-ab in serum. The remaining 11 patients were included > 6 months after onset of a currently monophasic disease course of LGI1-AE (P3-12, P15). For further clinical data at the time of the baseline PET-scans please refer to Table 2.

**Table 2:** Overview of LGI1-AE cohort at baseline [^18^F]F-DED PET scan

	P1		P2		P3		P4		P5		P6		P7		P8		P9		P10		P11		P12		P13		P14		P15	
**Clinical data**																														
Age, years	47		68		73		70		57		59		65		69		56		61		79		66		80		78		71	
Clinical status (maIM)	Improved after acute therapy, but impaired (0)		Improved after acute therapy, but impaired (1)		Slightly improved, cognitive impairment (3)		Slightly improved, cognitive impairment (11)		Not improved, cognitive impairment (12)		Slightly improved, cognitive impairment, psychiatric symptoms (16)		Improved, no symptoms (16)		Slightly improved, cognitive impairment, psychiatric symptoms (22)		Improved, cognitive impairment (37)		Slightly improved, cognitive impairment, psychiatric symptoms (32)		Improved, cognitive impairment (34)		Improved, cognitive impairment (43)		Clinical relapse with deterioration including cognitive impairment, psychiatric symptoms (76)		Clinical relapse with epileptic seizures, cognitive impairment (160)		Improved, cognitive impairment (177)	
mRS	3		3		2		2		2		2		0		4		2		3		1		1		3		3		3	
MoCA score (maIM)	23/30 (0)		20/30 (1)		26/30 (3)		29/30 (10)		23/30 (12)		28/30 (15)		na		23/30 (22)		30/30 (37)		24/30 (32)		na		30/30 (43)		23/30 (77)		17/30 (160)		24/30 (178)	
LGI1-Ab titer																														
Serum (maIM)	1:50 (0)		nd (3)		1:400 (3)		1:800 (10)		1:20 (12)		nd (15)		1:400 (16)		nd (22)		nd (33)		1:50 (32)		1:10 (34)		1:400 (43)		1:200(76)		1:50 (160)		1:50 (178)	
CSF (maIM)	na		na		1:1 (3)		nd (5)		nd (12)		nd (6)		na		nd (22)		nd (43)		nd (9)		nd (34)		na		1:5 (76)		na		na	
Other Ab titer (maIM)	Caspr2: 1:50 (0)																												NMDA nd (178)	
Immunotherapy	Corticosteroids PLEX IVIG Rituximab		Corticosteroids PLEX Rituximab		Corticosteroids PLEX		none		Corticosteroids		none		none		Rituximab		MTX		none		MTX		IVIG		Corticosteroids PLEX		Corticosteroids Rituximab		none	
**Imaging**																														
Anatomical side	right	left	right	left	right	left	right	left	right	left	right	left	right	left	right	left	right	left	right	left	right	left	right	left	right	left	right	left	right	left
**MRI**																														
MRI, maIM	0		0		3		14		7		16		14		20		34		26		33		45		76		161		177	
T2w/FLAIR hyperintens BG	-	-	-	-	-	-	-	-	-	-	-	-	-	-	-	-	-	-	-	+	-	-	+	-	-	+	-	-	-	-
T2w/FLAIR hyperintense MTL	+	-	+	+	-	-	+	-	+	-	-	-	-	-	+	+	+	+	+	+	-	+	+	+	-	-	+	+	+	-
Hippocampal volume	↔	↔	↔	↔	↔	↔	↔	↔	↔	↔	↔	↔	↔	↔	↔	↔	↔	↔	↓	↔	↓	↓	↔	↔	↔	↔	↔	↔	↔	↓
Whole brain volume	↔	↔	↔	↔	↔	↔	↔	↔	↔	↔	↔	↔	↔	↔	↔	↔	↔	↔	↔	↔	↓	↓	↔	↔	↓	↓	↔	↔	↔	↔
MTL volume	↓	↔	↓	↓	↑	↑	↓	↓	↑	↔	↔	↔	↔	↔	↓	↓	↓	↓	↔	↓	↓	↓	↑	↔	↓	↓	↑	↔	↓	↓
Gd-Enhancement	na	na	+	+	-	-	-	-	na	na	-	-	na	na	na	na	-	-	-	+	-	-	-	-	-	-	-	-	-	-
**1st [**^**18**^ **F]F-DED PET scan**																														
PET scan, maIM	0		0		3		11		12		16		16		22		33		35		36		44		77		163		181	
Visual read	+++		+++		+++		+		++		+++		+++		+++		++		+++		++		+++		+		++		+	

Ab: antibody; BG: basal ganglia; CASPR2: Contactin-associated protein-2; FLAIR: Fluid-attenuated inversion recovery; Gd: Gadolinium; IM initial manifestation;IVIG: intravenous immunoglobuline; maIM: months after initial manifestation; MoCA: Montreal-Cognitive-Assessment; MTL: mesio temporal lobe; MTX:methotrexate; na: not available; nd: not detectable; NMDA: N-Methyl-D-Aspartat. ↑ = increased; ↔ = normal; ↓ = reduced; + = abnormal; - = normal. +, ++,+++ indicates mild, moderate, and severe elevation of [18F]F-DED PET signals in the MTL as compared to controls in a visual read

### Cross-sectional [^18^F]F-DED-PET imaging for assessment of reactive astrogliosis in patients with LGI1-AE

First, we analyzed dynamic imaging data to determine a suitable reference tissue for simplified quantification and to evaluate the feasibility of [^18^F]F-DED assessment by late static imaging. Kinetic modelling of the full dynamic [^18^F]F-DED data was available for 14 patients with LGI1-AE and 11 controls. Quantification was performed using the previously established 1TC2k model for the full 60 min PET scan (Supplement Fig. 1A). The straight gyrus emerged as a suitable reference region since no significant difference in [^18^F]F-DED VTs was detected between patients with LGl1-AE and controls (*p* =.35). Despite the expected large variance of VTs at the group level, patients and controls showed similar distribution of individual VTs in the straight gyrus (Supplement Fig. 1B). Furthermore, 30–60 min [^18^F]F-DED SUVr referenced to the straight gyrus correlated with VT (ρ = 0.49; *p* <.001) and strongly with VTr (ρ = 0.91; *p* <.001) across multiple target regions (Supplement Fig. 2A and B). Agreement between SUVr and VTr are shown in Supplement Fig. 2C. In addition, reference tissue modeling yielded a sufficient fit in 22/25 cases with dynamic acquisition. DVR values showed good correlation with VTr/SUVr, reflecting consistent group differences (Supplement Fig. 3). As there was no correlation between SUVr and the age or gender, no further adjustment was made with this regard (Supplement Fig. 4).

**Fig. 1:**
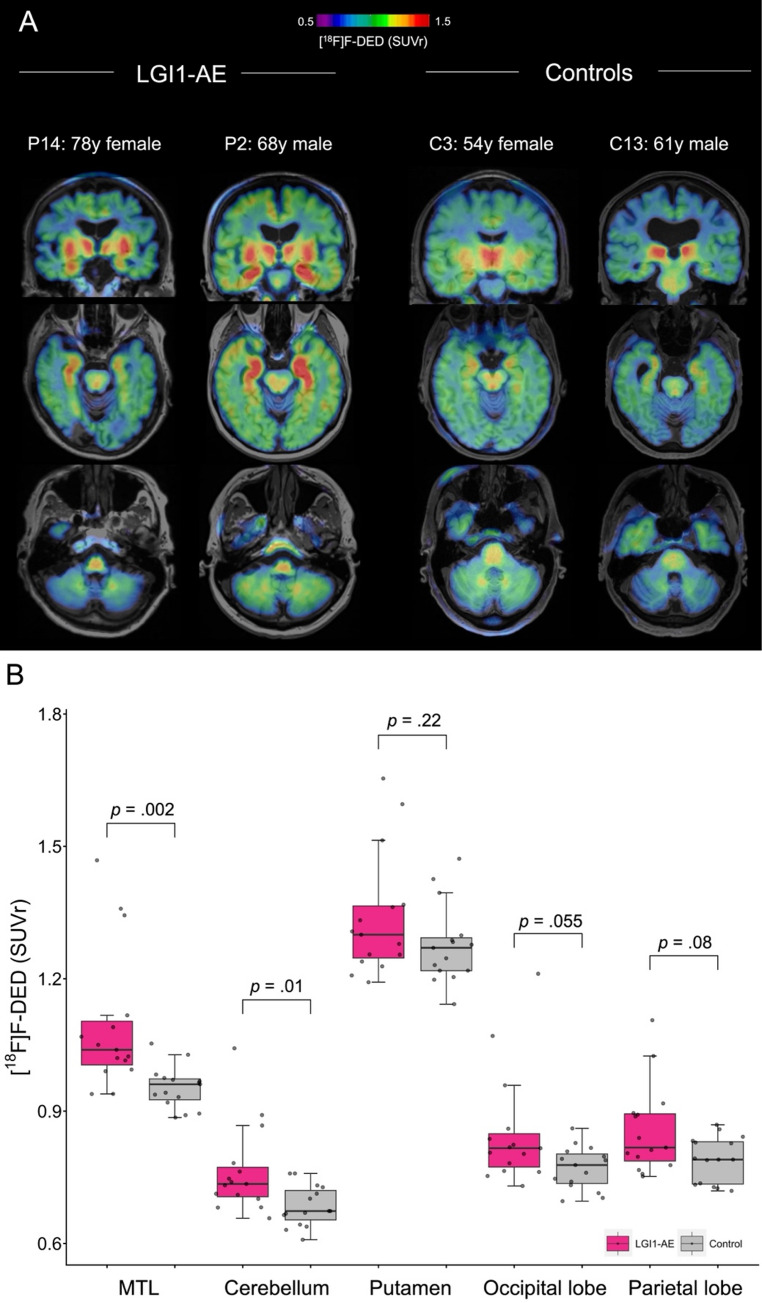
[^18^ F]F-DED PET detects astrogliosis in LGl1-AE patients **(A)** Coronal and axial planes of [^18^F]F-DED PET images (SUVr, referenced to straight gyrus) fused with individual MRI (T1 weighted) show two examples of LGI1-AE patients (mild and severe PET signal alteration) and two controls. All 15 LGI1-AE patients and 15 controls are displayed in the Supplement. **(B)** Quantitative [^18^F]F-DED PET SUVr (referenced to straight gyrus) in comparison of 15 LGI1-AE patients and 15 controls in different regions of interest: medial temporal lobe (MTL), cerebellum, putamen, occipital lobe and parietal lobe. Wilcoxon rank-sum test. FDR correction for *n* = 5 target regions

Second, to test weather astroglia PET has the capacity to detect disease activity in patients with LGI1-AE we performed visual and quantitative assessment of [^18^F]F-DED PET scans (30–60 min, SUVr). Visual analysis indicated mild (3/15), moderate (4/15) and severe (8/15) elevation of [^18^F]F-DED binding in the MTL of patients with LGl1-AE compared to controls (Table 1). Other brain regions did not show focal differences between patients and controls, but global signal elevation in cortical and subcortical brain regions was observed in several LGl1-AE patients (Fig. 1A, Supplemental Fig. 5). On the quantitative level, [^18^F]F-DED SUVr was significantly increased in the MTL (SUVr: 1.10 ± 0.16 vs. 0.95 ± 0.05; *p* =.002) as well as in the cerebellum (0.76 ± 0.10 vs. 0.68 ± 0.05; *p* =.014) and close to significance in the occipital (0.85 ± 0.13 vs. 0.77 ± 0.05; *p* =.055) and parietal lobes (0.86 ± 0.10 vs. 0.79 ± 0.05; *p* =.08) of LGI1-AE patients compared to controls (Fig. 1B). [^18^F]F-DED SUVr in the putamen (*p* =.22) was not significantly different between LGI1-AE patients and controls.

### Serial [^18^F]F-DED-PET imaging for monitoring the disease course of LGI1-AE

**Fig. 2:**
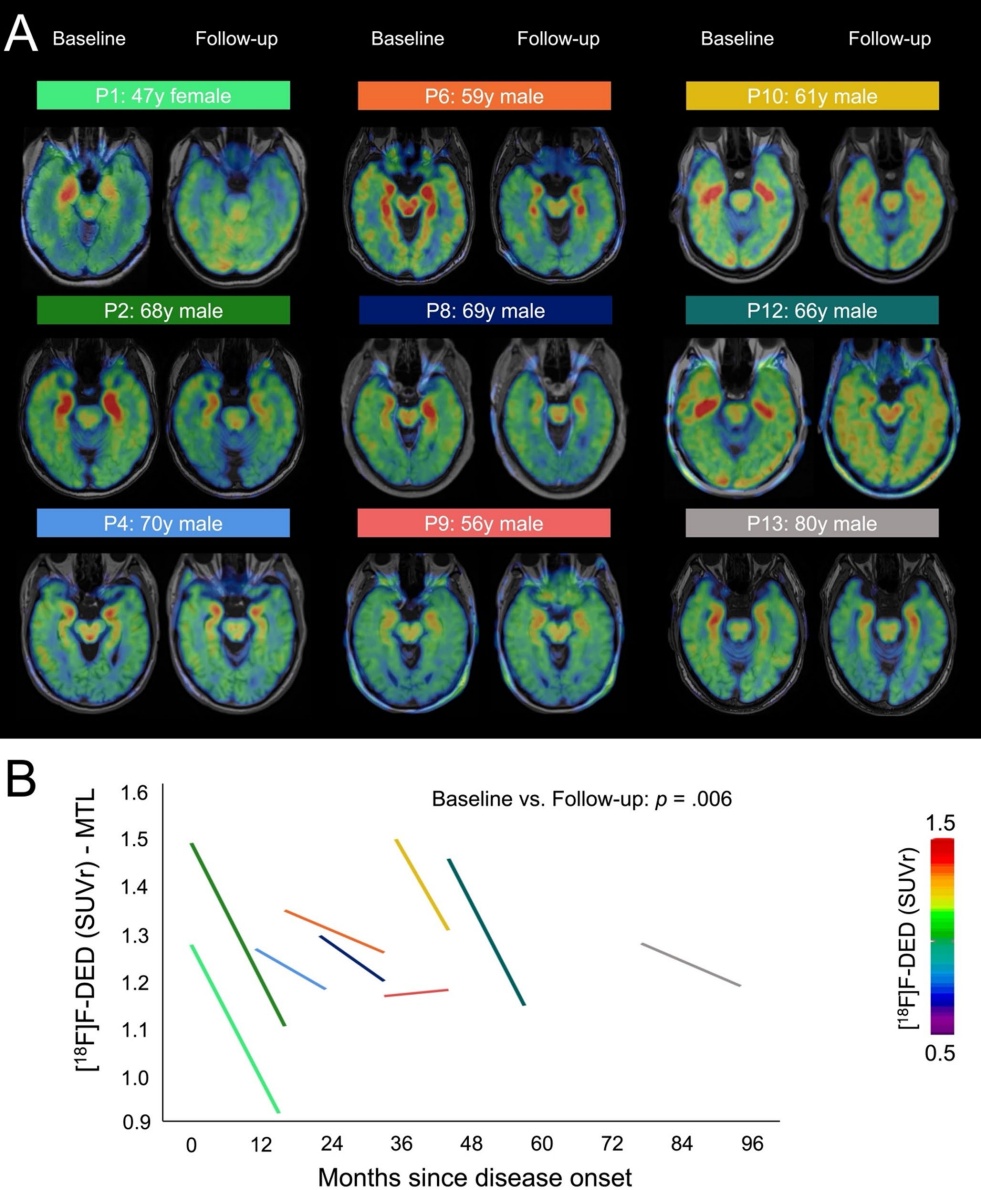
[^18^ F]F-DED PET allows monitoring of astrogliosis **(A)** Axial planes of [^18^F]F-DED PET images (SUVr, referenced to straight gyrus) fused with individual MRI (T1 weighted) of nine LGI1-AE patients comparing baseline to follow-up scan. **(B)** Quantitative [^18^F]F-DED PET SUVr (referenced to straight gyrus) of nine LGI1-AE patients comparing baseline and follow-up signals in the medial temporal lobe (MTL) (manual delineation of elevated regional signal at baseline). Two-sided paired t-test (*p* =.006)

Next, we evaluated if serial astroglia PET imaging allows monitoring of disease activity in LGI1-AE. Nine LGI1-AE patients underwent a follow-up PET examination (Table 3). P10 suffered a clinical relapse (mRS score from 3 to 4) with improvement after treatment with corticosteroids and plasma exchange, PET imaging was performed after acute relapse therapy. The remaining 8/9 patients were clinically stable or improved during the observation period. [^18^F]F-DED signals in the MTL were significantly reduced in the follow-up PET scans compared to baseline (1.35 ± 0.12 vs. 1.17 ± 0.11; *p* =.006), when delineating elevated regional signal at baseline manually (Fig. 2A). This finding was confirmed by the predefined MTL region of interest (Supplement Fig. 6). 8/9 patients revealed a decrease of the [^18^F]F-DED signal in the MTL, which was quantified consistent with the visual image impression (Fig. 2A and B; Table 1).

**Table 3:** Overview of LGI1-AE patients with a follow-up [^18^F]F-DED PET scan

	P1		P2		P4		P6		P8		P9		P10		P12		P13	
**Clinical data**																		
Age, years	49		69		71		60		71		57		62		67			
Clinical status (maIM)	Improved, cognitive impairment (17)		Improved, slight cognitive impairment (16)		Improved, slight cognitive impairment (17)		Stable, cognitive impairment, psychiatric symptoms (33)		Improved, slight cognitive impairment (38)		Improved, slight cognitive impairment (43)		Chronic clinical deterioration with cognitive impairment and psychiatric symptoms, improved after corticosteroids (45)		Improved, slight cognitive impairment (55)		Improved, cognitive impairment (90)	
mRS	2		1		1		2		1		2		4		0		1	
MoCA score (maIM)	23/30 (17)		26/30 (16)		na		28/30 (33)		na		30/30 (43)		21/30 (45)		26/30 (55)		29/30 (93)	
LGI1-Ab titer																		
Serum (maIM)	1:50 (17)		1:50 (16)		1:800 (17)		1:10 (33)		1:400 (38)		1:10 (43)		1:10 (43)		1:100 (55)		1:10 (93)	
CSF (maIM)	na		na		na		na		na		nd (43)		nd (43)		na		na	
Immunotherapy	Rituximab		Rituximab		none		none		none		MTX		Corticosteroids		Rituximab		Rituximab	
**Imaging**																		
Anatomical side	right	left	right	left	right	left	right	left	right	left	right	left	right	left	right	left	right	left
**MRI**																		
MRI, maIM	10		17		23		34		38		45		43		54		93	
T2w/FLAIR hyperintens BG	-	-	-	-	-	-	-	-	-	-	-	-	-	-	-	-	-	+
T2w/FLAIR hyperintense MTL	+	+	+	-	-	-	-	-	+	+	+	+	+	+	+	-	-	-
Hippocampal volume	↔	↔	↔	↔	↔	↔	↔	↔	↔	↔	↔	↔	↔	↔	↔	↔	↔	↔
Whole brain volume	↔	↔	↔	↔	↔	↔	↔	↔	↔	↔	↔	↔	↔	↔	↔	↔	↓	↓
MTL volume	↑	↑	↓	↓	↑	↓	↓	↓	↓	↓	↑	↓	↑	↓	↑	↔	↓	↓
Gd-Enhancement	na	na	-	-	na	na	na	na	-	-	-	-	-	-	-	-	-	-
**2nd [**^**18**^**F]F-DED PET scan**																		
PET scan, maIM	16		17		23		33		45		44		45		57		94	
Visual read	++		+		+		++		+		-		+		-		+	

Ab: antibody; BG: basal ganglia; FLAIR: Fluid-attenuated inversion recovery; Gd: Gadolinium; IM initial manifestation; maIM: months after initial manifestation; MoCA: Montreal-Cognitive-Assessment; MTL: mesio temporal lobe; MTX: methotrexate; na: not available; nd: not detectable. ↑ = increased; ↔ = normal; ↓ = reduced; + = abnormal; - = normal. -, +, ++ indicates none, mild, and moderate elevation of [^18^F]F-DED PET signals in the MTL as compared to controls in a visual read

### Asymmetry of [^18^F]F-DED binding in the MTL

**Fig. 3:**
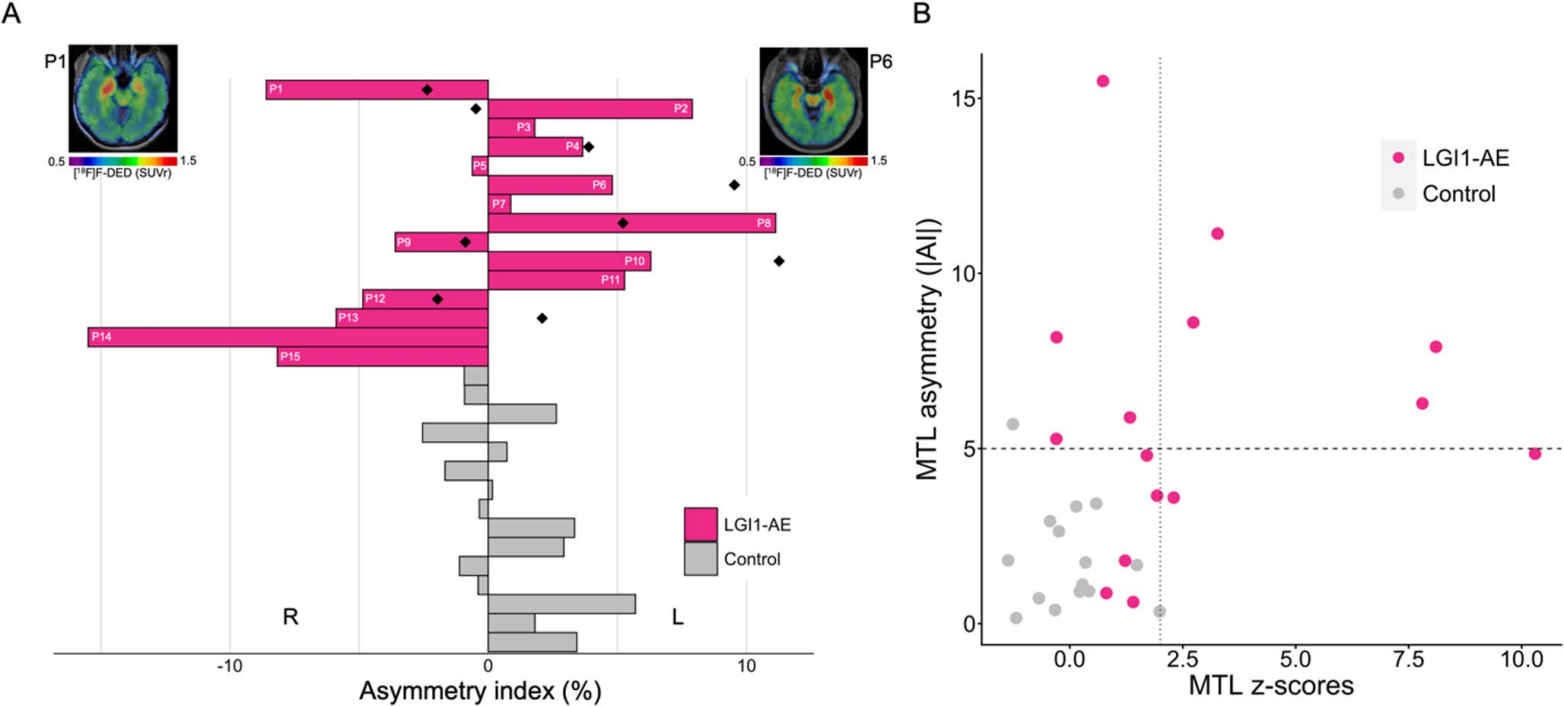
Asymmetry of [^18^ F]F-DED PET signals in the MTL of LGl1-AE patients **(A)** Assessment of mesial temporal asymmetry in [^18^F]F-DED PET images of 15 LGI1-AE patients compared to 15 controls via calculation of the asymmetry index: AI = [200x(L-R)/(L + R)] %. Wilcoxon rank-sum test (*p* <.001). Diamonds representing mesial temporal asymmetry of follow-up LGI1–AE patients and comparing them to baseline asymmetry. Two-sided paired t-test (*p* =.2). Representative [^18^F]F-DED PET images (SUVr, referenced to straight gyrus) show exemplary LGI1-AE patients with strong asymmetry in right and left mesial temporal lobe R = right, L = left, P = patient number, Diamonds = follow-up patients. **(B)** Scatter plot showing individual z-scores of [^18^F]F-DED PET SUVr (referenced to the straight gyrus) values in the mesial temporal lobe (MTL), plotted against the asymmetry index (|AI|) of LGI1-AE and control patients The vertical dotted line indicates the pathological threshold for MTL z-scores (≥ 2), while the horizontal dashed line marks the pathological threshold for asymmetry (|AI| >5%)

Based on the observation of asymmetric focal tracer uptake in the MTL, we asked if an analysis of signal symmetry could further improve the detection of astrogliosis in LGl1-AE. To this end, individual AI were calculated for MTL [^18^F]F-DED SUVr (Fig. 3A). Here, 8/15 patients with LGI1-AE showed a unilaterally emphasized uptake (asymmetry index > 5%), whereas only 1/15 of the controls had an asymmetry index > 5% (*p* <.001). At group level, the AI decreased in the follow-up examination compared to baseline PET without reaching significance (6.30 ± 2.50; 4.19 ± 3.83; *p* =.20). We evaluated if asymmetric MTL tracer uptake could provide additional diagnostic value in patients with LGl1-AE and found a high AI also in 4 patients with moderate overall MTL [^18^F]F-DED PET SUVr (z-score < 2) (Fig. 3B).

### Astroglia PET as a complementary read-out to MRI and clinical data in LGI1-AE

**Fig. 4:**
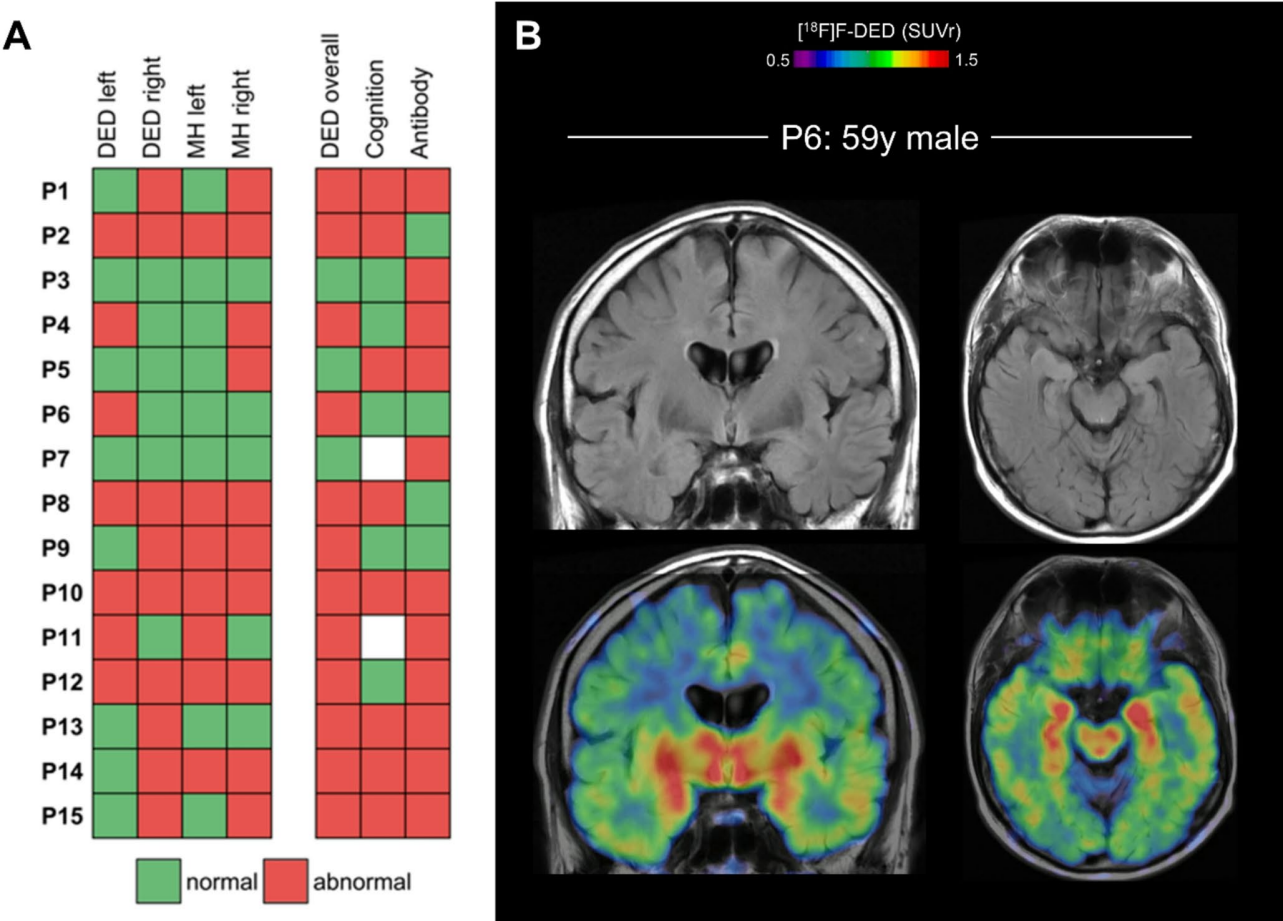
Complementary information of [^18^ F]F-DED PET signals to routine clinical data **(A)** Heatmap of LGI1-AE patient data, showing normal and abnormal findings for [^18^F]F-DED uptake and MTL hyperintensity in the left and right MTL on MRI. Patients with abnormal [^18^F]F-DED uptake in either MTL were classified as having abnormal [^18^F]F-DED (DED overall). For cognition a MoCA < 26 was considered abnormal. Antibody status was defined as detectable versus not detectable. MH = medial temporal lobe hyperintensity. **(B)** Example of an individual LGl1-AE patient (P6) with elevated MTL [^18^F]F-DED binding but unsuspicious MRI

Finally, we evaluated if astroglia PET indices provide complementary information in LGl1-AE compared to current standard of care. Noteworthy, in two patients (P6, P13), the MTL was visually assessed as unremarkable in the T2/FLAIR MRI sequence, but the PET examination revealed astrogliosis with clearly abnormal tracer binding on visual and quantitative level (z-score > 2) (Fig. 4A). One patient had T2/FLAIR hyperintensity in the MTL that was not associated with an increased z-score or significant asymmetry index in the PET scan (P5). Two patients showed unremarkable presentations in MRI and PET, whereas for the other patients, there was concordance between abnormal MRI and abnormal PET findings in the MTL (Fig. 4B). In terms of clinical severity, neither the mRS scores nor the MoCa tests at the time of baseline PET scans showed quantitative association with the [^18^F]F-DED signals in the MTL (ρ = 0.23; *p* =.41; respectively ρ = 0.18, *p* =.57) (Supplement Fig. 7A, 5B).

## Discussion

In this pilot cohort of 15 LGI1-AE patients and 15 controls, we investigated the novel MAO-B PET radiotracer [^18^F]F-DED for assessment of reactive astrogliosis as an index of disease activity. Moderate to strong PET signals with predominance in the MTL allowed discrimination from controls on visual and quantitative levels. Additionally, follow-up imaging of 9 LGI1-AE patients revealed decreasing [^18^F]F-DED PET signals over time, showing the ability to monitor the evolution of astrogliosis during the LGI1-AE disease course. Notably, our analysis revealed a marked asymmetry of [^18^F]F-DED PET signals. Finally, we observed that [^18^F]F-DED PET signals provide a complementary read-out compared to current standard of care.

First, in the cross-sectional analysis using [^18^F]F-DED PET, patients with LGI1-AE revealed increased MAO-B expression in the MTL compared to controls. The findings regarding the MTL are consistent with histopathologic studies of LGI1-AE brain tissue, although only limited clinical and preclinical data are available in this rare disease: Recently, hippocampal astrocyte activation was reported in a rat model of AE (with infusion of purified human LGI1-ab) [23]. Further, reactive activation of astrocytes in AE was also shown in human tissue, especially in the hippocampus, but not in other cortical areas using GFAP staining [14–16]. However, previous PET studies reported on higher MAO-B expression especially compared to the occipital and parietal cortical areas in their control cases [20, 24]. We therefore emphasize the importance of the SUVr visualization as well as the asymmetry analysis for the assessment of the [^18^F]F-DED uptake in the MTL. Our data is in line with previous imaging results: (i) MRI studies have repeatedly shown that a T2w/FLAIR hyperintense MTL is an important imaging marker for LGI1-AE [21]. Different studies were performed to biologically characterize hyperintensities, which represent an unspecific index, in more detail by use of various radiotracers. (ii) [^18^F]FDG PET imaging repeatedly showed altered glucose uptake in the MTL [6], potentially driven by increased glucose uptake of immune cells [25]. Increased 18 kDa translocator protein (TSPO) expression in the MTL as well as in the frontal cortex was reported ([^18^F]DPA-714 PET) [10, 26]. Interestingly, our cohort did not show elevated frontal cortex MAO-B PET signals neither at the group level nor at the individual level. Thus, expression patterns of TSPO and MAO-B may not entirely overlap in LGl1-AE. While TSPO is upregulated in both microglia and astrocytes, a high correlation between GFAP-positive astrocytes and MAO-B expression has been reported using immunohistochemistry of mouse tissue [17]. In neurodegenerative diseases, MAO-B expression has been described as an early biomarker in the disease course [27], highlighting its potential to uncover distinct neuroinflammation profiles compared to TSPO. Additional neuropathological investigations are necessary to elucidate the role of MAO-B in LGI1-AE within this context. Further, we detected an increased MAO-B expression in the cerebellum within the LGI1-AE cohort compared to controls. Possible astroglial pathologies in the cerebellum in LGI1-AE should be investigated in more detail in the future, as the cerebellum is a rather atypical predilection site in LGI1-AE. Overall, our results provide evidence that astrogliosis can be detected in LGI1-AE patients in vivo when applying [^18^F]F-DED PET to target MAO-B. In this regard, first-in-human imaging and preclinical characterization of [^18^F]F-DED has shown broad opportunities of application for this radiotracer and revealed MAO-B positive astrocytes as the major source of PET signal increases [17, 28]. Analysis of this cohort of LGI1-AE patients that received [^18^F]F-DED PET suggests that simplified imaging of a short time window (30–60 min p.i.) allows robust detection of focal MTL signals by visual and quantitative approaches, validated against previously established kinetic modeling with image derived input function [17]. We note that the applied kinetic modelling approaches were only evaluated in a limited number of human scans for this tracer so far [17] and deserve validation against arterial sampling. Thus, we focused on a simplified clinical approach via scaling of SUV images which can be implemented in molecular imaging units with routine software. Our rationale was to provide a tool to neurologists and nuclear medicine physicians that can be used at different centers at low frequency due to the rare character of the disease and with low-cost setup. Taken together, our observations indicate that the application of this radiotracer for assessment of astrogliosis in AE can also be established in routine settings at tertiary centers.

Second, we were able to show that serial [^18^F]F-DED PET allows monitoring of LGI1-AE disease activity, since decreases of MTL PET signals were observed during clinical stabilization in the course of the disease in most patients. Interestingly, we observed two different patterns of [^18^F]F-DED signal decline in the MTL: 4/9 patients, including those scanned in the acute phase, showed a rapid decline, whereas 5/9 showed a comparably slower decline. However, this preliminary observation is currently limited mainly due to the size of this subgroup and needs further conformation with close evaluation of influencing factors. As a limitation of the current study, no meaningful comparison could be made between untreated patients and patients under immunotherapy, due to the small and heterogeneous cohort. However, similar results, reporting a decrease in pathological tracer binding in the MTL during the course of the disease, were also reported using [^18^F]FDG-PET as a less specific index [29]. In future studies, consideration of longer follow-up periods could allow for detection of larger changes in astroglial activity compared to the current setup with an average follow-up period of 15 months.

Third, we could show that the majority of LGI1-AE patients have an asymmetry in tracer binding in the MTL, which we interpret as predominant astroglial disease activity of one hemisphere. On the group level, this asymmetry also appeared to regress during the course of the disease, supporting the validity of this biologically interesting finding. Using cluster analysis, we demonstrate that using an asymmetry index of > 5% and an MTL SUVr z-score of > 2 LGI1-AE patients may aid to improve the discrimination from controls. Interestingly, a previous study using [^18^F]FDG-PET, a side asymmetry in tracer uptake was also detected in LGI1-AE patients [30], again confirming biological asymmetry in vivo. As the potential underlying correlate, a significant different LGI1 protein expression between both sides could be detected in a small subgroup with post-mortem brain analyses [30].

Fourth, we correlated the [^18^F]F-DED PET data with clinical and MRI data at baseline examination to explore if astroglia PET provides additional information compared to current standard of care. There was no correlation between astrogliosis and mRS- or MoCA-scores, suggesting that [^18^F]F-DED PET signals do not simply reflect clinical severity. We note that this result could also relate to the small sample size, heterogenous timepoints of examinations and unspecific clinical scores for autoimmune encephalitis. One previous MRI study has reported a correlation between signal intensity in the MTL and basal ganglia based on T2 and DWI sequences and the mRS score in LGI1-AE [31], while no larger studies correlated mRS or MoCA scores with PET read-outs. In addition, we observed a concordance between topology in MRI and PET with the MTL as the predilection site that showed concordance of abnormality in MRI and PET in most individuals. However, in two cases, PET showed a diagnostic advantage since MTL regions revealed an increased [^18^F]F-DED uptake in the MTL while the MRI was unremarkable. Furthermore, [^18^F]F-DED PET signals were increased not only in the MTL but also in the cerebellum, which could indicate another predilection site of astroglial disease activity in LGI1-AE that is more difficult to assess with MRI. Still, we note that we did not intend to perform a head-to-head comparison between PET and MRI, since different MRI protocols precluded a reliable qualitative and quantitative evaluation.

Limitations of this study result from the small number of patients examined, the reduced number of patients in the longitudinal analysis as well as the cross-sectional monocentric study design. Thus, we kept the different individual patient courses descriptive and did not investigate PET signal differences related to co-factors such as disease duration, reactivation, and immunotherapy. Nonetheless, our pilot findings in individual patients with LGI1-AE encourage systematic larger cohort studies with [^18^F]F-DED PET imaging and external validation. The qualitative MRI assessment represents a further limitation. Longitudinal studies with larger cohorts should therefore include standardized MRI protocols to allow a head-to-head comparison of changes in astroglia PET and MRI. Furthermore, physiological MAO-B expression, which is highest in the basal ganglia (i.e. caudate nucleus and thalamus) and lowest in the cerebellum, but also shows higher levels in the MTL compared to neocortical structures [20], needs to be considered during interpretation of [^18^F]F-DED PET scans.

In conclusion, this is the first report on the use of [^18^F]F-DED PET in a patient cohort with autoimmune-encephalitis. Here we show that [^18^F]F-DED-PET can be a promising biomarker to evaluate and monitor in vivo astrogliosis in patients with LGI1-AE from a biological and clinical perspective.

## Supplementary Information

Below is the link to the electronic supplementary material.

Supplementary Material 1 (DOCX 8.81 MB)

## Data Availability

Anonymized data not published within this article will be made available by request from any qualified investigator.
