# Supplementary material for: [18F]fluorodeprenyl-D2 PET can detect and monitor astrogliosis in anti-LGI1-IgG autoimmune encephalitis
Source: Eur J Nucl Med Mol Imaging. 2025 Sep 26;53(3):2054–68. doi: 10.1007/s00259-025-07531-5 (PMC12860853; doi:10.1007/s00259-025-07531-5)
Supplement: Supplementary file 1 — Supplementary Material 1 (DOCX 8.81 MB) [file 259_2025_7531_MOESM1_ESM.docx]

**Supplementary Material**

|  | **Age at PET-scan** | **Gender** | **Diagnosis** | **MMSE** | **β-amyloid PET** |
| --- | --- | --- | --- | --- | --- |
| C1 | 59 | F | Oligodendroglioma WHO II°, right frontal | NA | NA |
| C2 | 28 | F | Healthy control | NA | NA |
| C3 | 54 | F | Subjective cognitive impairment | MMSE: 30 | negative |
| C4 | 77 | M | Subjective cognitive impairment | MMSE: 30 | negative |
| C5 | 81 | M | Subjective cognitive impairment | NA | negative |
| C6 | 59 | F | Subjective cognitive impairment | MMSE: 30 | negative |
| C7 | 85 | F | Subjective cognitive impairment | MMSE: 28 | negative |
| C8 | 80 | M | Subjective cognitive impairment | MMSE: 30 | negative |
| C9 | 74 | F | Subjective cognitive impairment | MMSE: 29 | negative |
| C10 | 76 | F | Subjective cognitive impairment | NA | negative |
| C11 | 79 | F | Subjective cognitive impairment | MMSE: 29 | negative |
| C12 | 72 | M | Subjective cognitive impairment | NA | negative |
| C13 | 61 | M | Subjective cognitive impairment | MMSE: 28 | negative |
| C14 | 80 | M | Subjective cognitive impairment | NA | negative |
| C15 | 79 | M | Subjective cognitive impairment | MMSE: 29 | negative |

**Supplement Table 1 Demographic data of the control cohort**

MMSE = Mini Mental State Examination. F = female; M = male. NA = not available


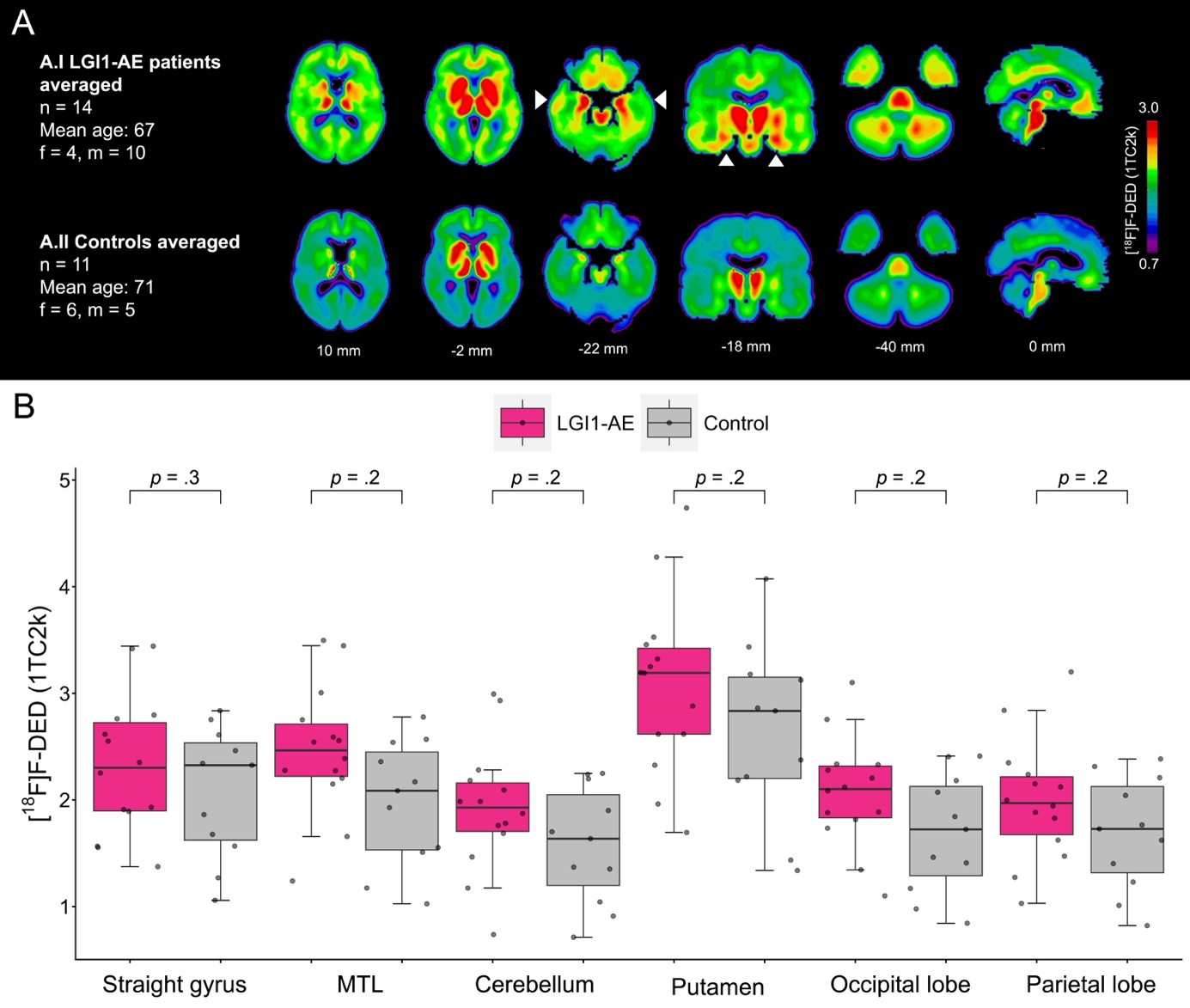


**Supplement Fig. 1 Validation of the straight gyrus as a reference tissue for [^18^F]F-DED PET imaging in LGI1-AE**

**(A)** Axial, coronal and sagittal planes show averaged [^18^F]F-DED volumes of distribution (VT) derived from the 1TC2k compartment model at levels of neocortical regions, basal ganglia, cerebellum (all axial), hippocampus (axial and coronal) and brainstem (sagittal). In **(A.I)** 14 1TC2k VT images of LGI1-AE patients and in **(A.II)** eleven 1TC2k VT images of controls were averaged. n = number of patients averaged, f = female, m = male, triangles showing the medial temporal lobe as the region of interest

**(B)** Quantitative [^18^F]F-DED (1TC2k) comparing 14 LGI1-AE patients to 11 controls in multiple target regions such as frontal lobe straight gyrus, medial temporal lobe (MTL), cerebellum, putamen, occipital lobe and parietal lobe. P-values were calculated using independent two-sample t-tests. FDR correction was applied.


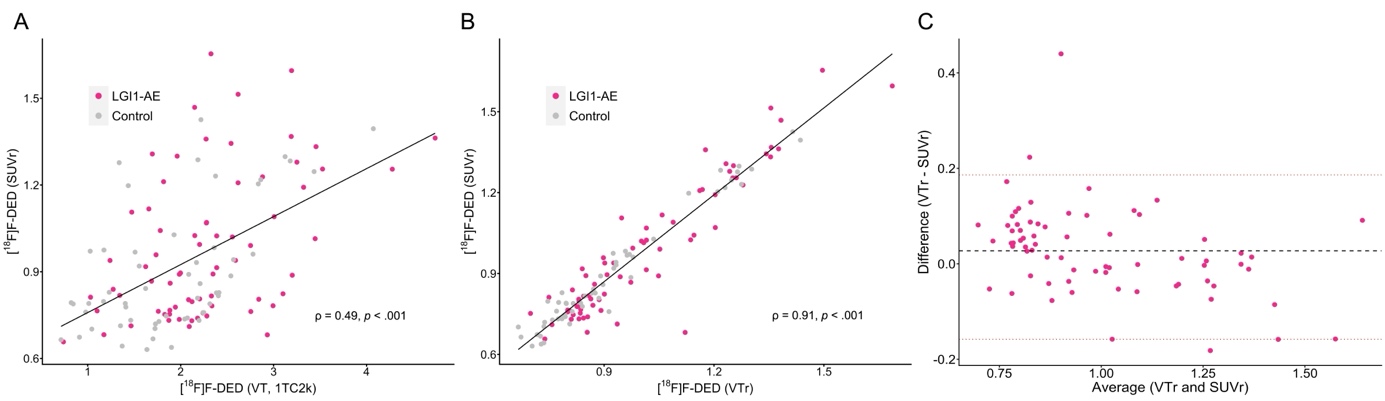


**Supplement Fig. 2 Validation of simplified PET quantification against kinetic modelling with image-derived input functions**

**(A)** Correlation between [^18^F]F-DED PET SUVr (referenced to straight gyrus) and [^18^F]F-DED PET VT (derived from 1TC2k model) tested in multiple target regions of 14 LGI1-AE patients and eleven control patients. The results show a Spearman correlation coefficient of ρ = 0.49 with *p* < .001.

**(B)** Correlation between [^18^F]F-DED PET SUVr (referenced to straight gyrus) and [^18^F]F-DED PET VTr (derived from 1TC2k model, referenced to straight gyrus) tested in multiple target regions of 14 LGI1-AE patients and eleven control patients. The results indicate a strong positive correlation, with Spearman‘s ρ = 0.91 with *p* < .001.

**(C)** Bland-Altman plot comparing VTr and SUVr values. Since the distribution of the differences was not normal (Shapiro–Wilk test: *p* < .001), non-parametric statistics were used. The median difference (bias) was 0.027 (black dashed line), with an interquartile range (IQR) of 0.11. The non-parametric 95% limits of agreement were estimated as the 2.5th and 97.5th percentiles, ranging from –0.16 to 0.19 (red dotted lines).

**
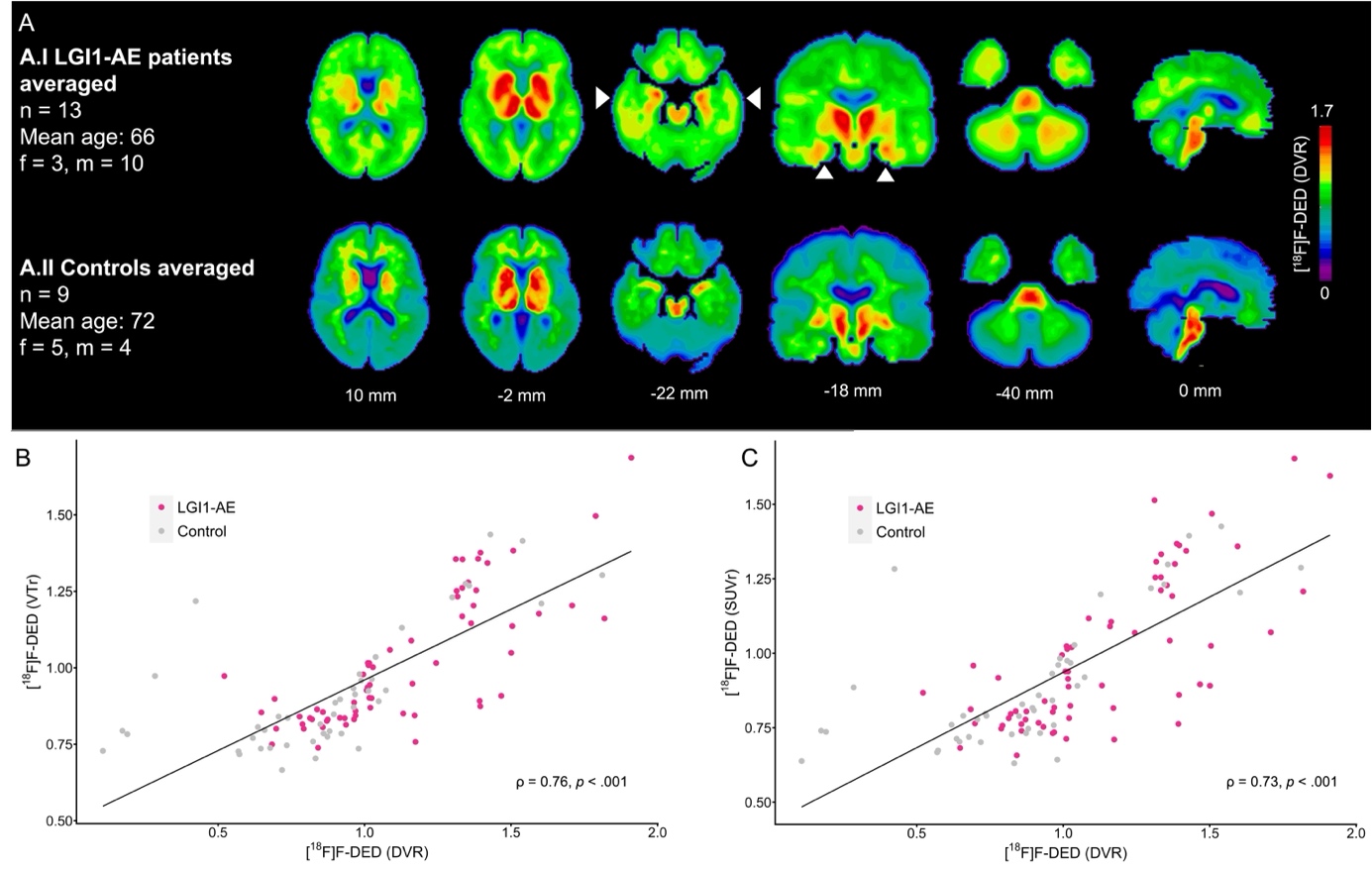
**

**Supplement Fig. 3 Validation of simplified [^18^F]F-DED PET quantification using SUVr and VTr against BPnd**

**(A)** Axial, coronal and sagittal planes show averaged [18F]F-DED PET DVR (Distribution Volume Ratio) (derived from BPnd model using straight gyrus as a reference region) at levels of neocortical regions, basal ganglia, cerebellum (all axial), hippocampus (axial and coronal) and brainstem (sagittal). In (A.I) 13 DVR images of LGI1-AE patients and in (A.II) nine DVR images of control patients were averaged.

n = number of patients averaged, f = female, m = male, triangles showing the mesiotemporal lobe as the region of interest

**(B)** Correlation between [18F]F-DED PET DVR (straight gyrus as a reference region) and [18F]F-DED PET VTr (referenced to straight gyrus) tested in multiple target regions of 13 LGI1-AE patients and nine control patients. The results indicate a positive correlation, with Spearman‘s ρ = 0.76 with p < 2.2e-16).

**(C)** Correlation between [18F]F-DED PET DVR (straight gyrus as a reference region) and [18F]F-DED PET SUVr (referenced to straight gyrus) tested in multiple target regions of 13 LGI1-AE patients and nine control patients. The results indicate a positive correlation, with Spearman‘s ρ = 0.73 with p < 2.2e-16).


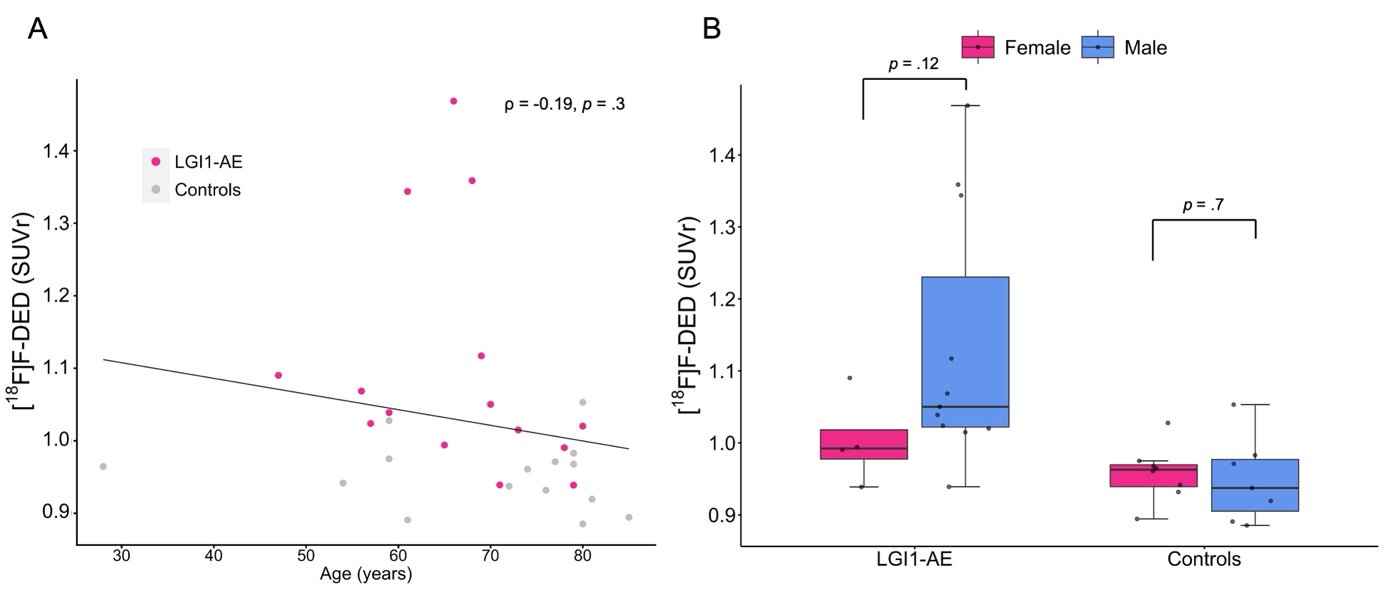


**Supplement Fig. 4 Analysis of [^18^F]F-DED PET SUVr in relation to age and sex in LGI1-AE patients and controls.**

**(A)** Correlation between [18F]F-DED PET SUVr (referenced to straight gyrus) and age tested in the region of interest: mesial temporal lobe (MTL) of 15 LGI1-AE patients and 15 Controls. The results show a Spearman correlation coefficient of ρ = -0.19 with p = 0.30.

**(B)** Comparison of [18F]F-DED PET SUVr (referenced to straight gyrus) values between female and male LGI1-AE patients and Controls in the region of interest: mesial temporal lobe (MTL). P-value of LGI1-AE patients calculated using a Wilcoxon rank-sum test. P-value of Controls calculated using an independent two-sample t-test.

**Supplement Fig. 5 [^18^F]F-DED PET images of all individual LGl1-AE patients and controls at baseline**

Coronal and axial planes of [^18^F]F-DED PET images (SUVr, referenced to straight gyrus) fused with individual MRI (T1 weighted) of 15 LGI1-AE patients and 15 controls.


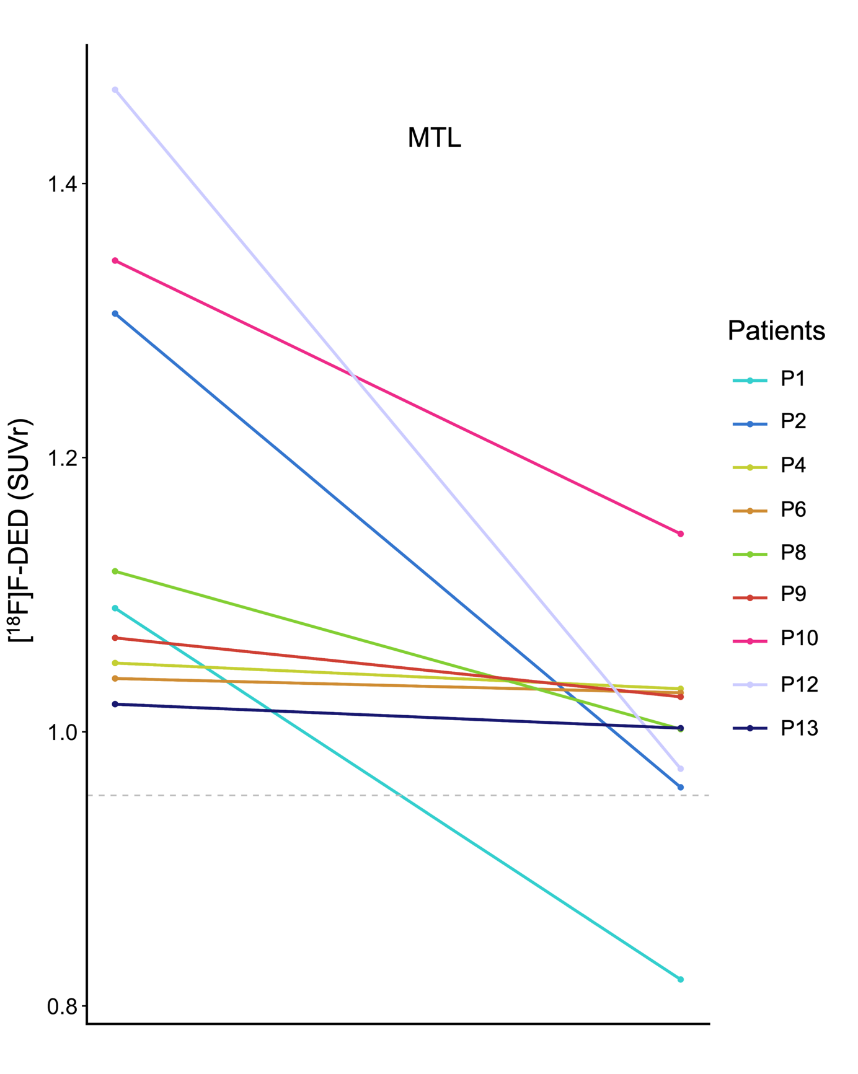


**Supplement Fig. 6 Validation of PET monitoring with predefined MTL regions of interest**

Quantitative [^18^F]F-DED PET SUVr (referenced to straight gyrus) of nine LGI1-AE patients comparing baseline and follow-up in the region of interest: medial temporal lobe (two-sided paired t-test, p = 0.019).

Grey line = mean [^18^F]F-DED PET SUVr (referenced to straight gyrus) in medial temporal lobe of controls

SUVr values were obtained using predefined regions of interest in PMOD.

**
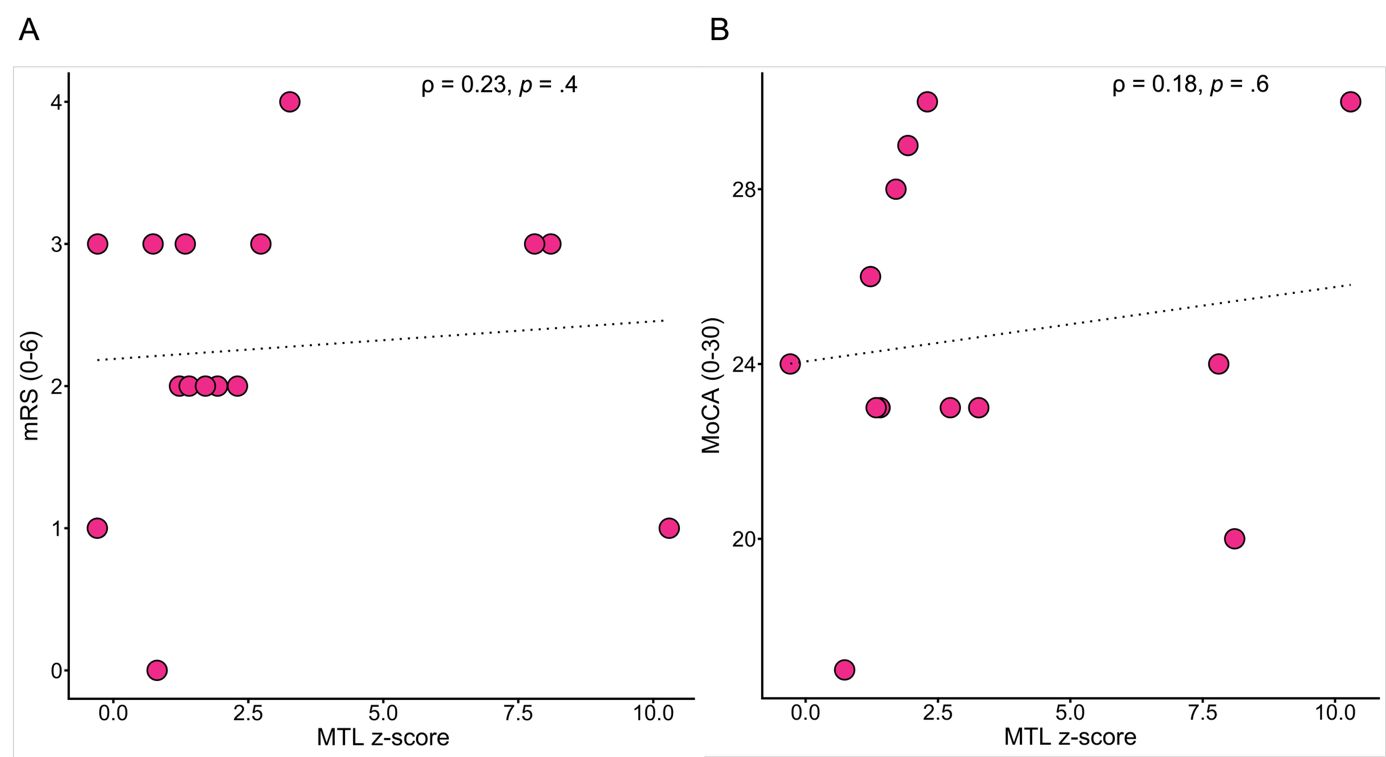
**

**Supplement Fig. 7 Complementary information of [^18^F]F-DED PET signals to clinical scores**

**(A)** The correlation between individual z-scores of SUVr values (referenced to the straight gyrus) in the mesial temporal lobe (MTL) and mRS (0-6) test results for 15 LGI1-AE patients yielded a Spearman correlation coefficient of ρ = 0.23 with *p* = .4.

**(B)** The correlation between individual z-scores of SUVr values (referenced to the straight gyrus) in the mesial temporal lobe (MTL) and MoCA (0-30) test results for 13 LGI1-AE patients showed a Spearman correlation coefficient of ρ = 0.18 with *p* = .6.
